# The Beat

**Published:** 2009-07

**Authors:** Erin E. Dooley

## FDA to Revisit BPA Decision

In June 2009, FDA Commissioner Margaret Hamburg announced the agency is revisiting its August 2008 decision that levels of bisphenol A (BPA) found in plastic bottles and other food containers are safe. Acting FDA Chief Scientist Jesse Goodman will lead the reevaluation, which should be completed by early fall 2009. Amidst the growing debate over the safety of BPA, several U.S. states and municipalities have enacted restrictions on the chemical, and some manufacturers and retailers have voluntarily removed the chemical from their products and shelves.

## Cutting Marine Litter

Marine litter poses complex environmental, economic, health, and aesthetic problems in water bodies around the world. In the April 2009 report *Marine Litter: A Global Challenge*, UNEP outlines methods to reduce this waste stream, such as the requirement that food vendors in national parks use biodegradable plates and cups, incentives for fishermen to remove debris from the ocean when they spot it, and taxes on plastic grocery bags. In Ireland such a tax led to a 90% reduced use of these items. The report also called for investment in waste management infrastructure and education and outreach to exchange technical information and generate a sense of environmental stewardship.

**Figure:**
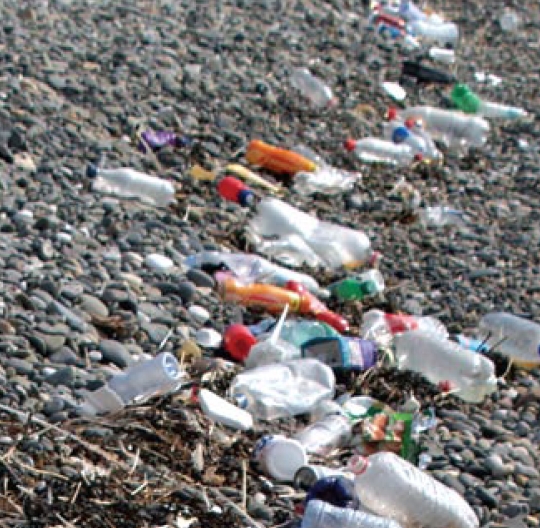
Marine litter affects all the oceans of the world.

## Smoking and Gum Disease: More to the Story

Smokers are more prone to chronic gum disease caused by the bacterium *Porphyromonas gingivalis* than nonsmokers and can have more severe symptoms and poorer response to conventional treatments. In the May 2009 issue of *Environmental Microbiology*, scientists show that exposure to cigarette smoke alters *P. gingivalis* genes associated with detoxification, oxidative stress mechanisms, and DNA repair. The resulting changes in the expression of proteins in cell membranes can affect how the smoker’s immune system reacts to the pathogen. This finding may help researchers find better treatments for *P. gingivalis* infections.

## Cellular Switch for Allergies, Asthma Discovered

Kelly Speiran et al. report in the May 2009 issue of the *Journal of Leukocyte Biology* the discovery of a mechanism that turns allergies and asthma on and off. When cytokines IL-4 and IL-10, which initiate immune responses, were added to developing mast cells, those cells died, leading the researchers to conclude that not only are the cytokines the “on switch” for immune response, they are also the “off switch” that stops overproliferation of mast cells, which can cause allergies and asthma. The authors wrote, “Our data support the theory that IL-4 and IL-10 function as endogenous regulators of mast cell development and are produced by immature mast cells during their ontogeny.” The team hopes their work will lead to new medications that treat the root causes of these diseases.

**Figure:**
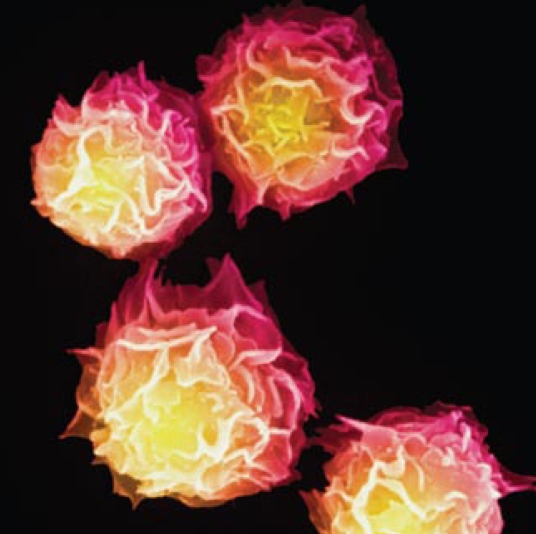
Mast cells play a central role in inflammatory and allergic reactions.

## Well Testing for Well Children

In May 2009 the American Academy of Pediatrics issued new guidelines, developed in conjunction with the NIEHS, recommending that private well owners test their drinking water at least once per year for contaminants such as nitrate, coliform bacteria, and metals including lead and arsenic. Well water should also be tested when a child is born or if the well sustains structural damage. Children can become dehydrated quickly if they develop diarrhea from drinking bacterially contaminated water, and nitrates can cause a potentially serious condition known as methemoglobinemia in infants. Private well water, which is used by 6% of households in the United States, is not subject to federal water regulations.

## Concrete’s Carbon Footprint

The concrete industry is believed to contribute about 5% of global CO_2_ emissions. Decades after it is laid, concrete—the world’s most commonly used building material—reabsorbs small amounts of CO_2_ to form calcite. Data published by Liv Haselbach in the June 2009 *Journal of Environmental Engineering* suggest concrete may form other carbon-based compounds besides calcite and, in the process, may reabsorb more CO_2_ than previously thought. A better understanding of the complexities of concrete’s reabsorption processes may help scientists develop new ways to speed up CO_2_ reabsorption in recycled concrete and pavement.

**Figure:**
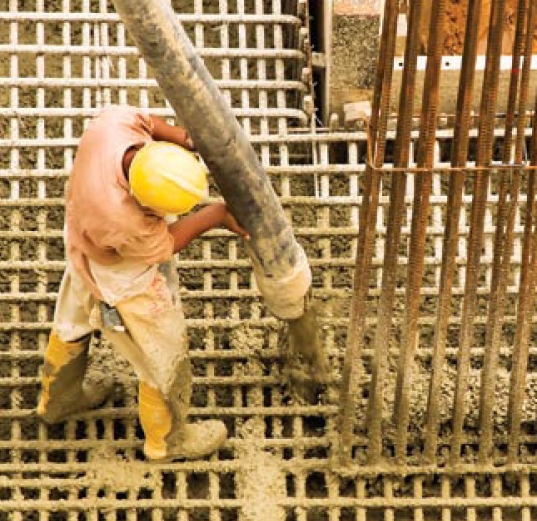
Concrete is the world’s most commonly used building material.

